# Self-assembly of peptide amphiphiles by vapor pressure osmometry and dissipative particle dynamics

**DOI:** 10.1039/c8ra04692a

**Published:** 2018-07-24

**Authors:** Taiga Seki, Noriyoshi Arai, Donguk Suh, Taku Ozawa, Tomoko Shimada, Kenji Yasuoka, Atsushi Hotta

**Affiliations:** Department of Mechanical Engineering, Keio University 3-14-1 Hiyoshi Kohoku-ku Yokohama 223-8522 Japan hotta@mech.keio.ac.jp; Department of Mechanical Engineering, Kindai University 3-4-1 Kowakae Higashiosaka Osaka 577-8522 Japan; Research Institute for Science and Technology, Kindai University 3-4-1 Kowakae Higashiosaka Osaka 577-8522 Japan arai@mech.kindai.ac.jp; Department of Mechanical Engineering, University of Tokyo 7-3-1 Hongo Bunkyo-ku Tokyo 113-8656 Japan; Materials Science Section, Engineering Technology Division, JSOL Corporation Harumi Center Bldg., 2-5-24, Harumi Chuo-ku Tokyo 104-0053 Japan; Asahi-Kasei Corporation 1-105 Jimbocho, Kanda Chiyoda-ku Tokyo 100-8101 Japan

## Abstract

Peptide amphiphiles are one of the most promising materials in the biomedical field, so much effort has been devoted to characterizing the mechanism of their self-assembly and thermosensitive gelation. In this work, vapor pressure osmometry measurements were carried out to parameterize the thermosensitivity of interactions between peptide amphiphiles in an aqueous solution. The osmometry measurement verified that the peptides became more hydrophobic as temperature increased, which was quantitatively described with the Flory–Huggins *χ* parameter. Thereafter, a coarse-grained molecular model was used to simulate peptide amphiphiles dissolved in an aqueous solution. The temperature sensitive coarse-grained parameter *a*_HW_, which is the repulsive force between the hydrophilic head of the peptide amphiphile and water was estimated from the aforementioned experimentally obtained *χ*. Furthermore, the effects of concentration and temperature on the self-assembly behavior of peptide amphiphiles were quantitatively studied by dissipative particle dynamics. The simulation results revealed that *a*_HW_ plays an important role in self-assembly characteristics and in the resulting microstructure of the peptide amphiphiles, which coincides with previous experimental and computational findings. The methodology in quantitatively linking the coarse-grained parameter from experiment and theory provides a sensible foundation for bridging future simulation studies with experimental work on macromolecules.

## Abstract 2

Vapor pressure osmometry measurements and molecular simulation were carried out to investigate the self-assembly behavior of peptide amphiphiles. The results revealed that the head–water interaction plays an important role for their microstructure.

## Graphical abstract

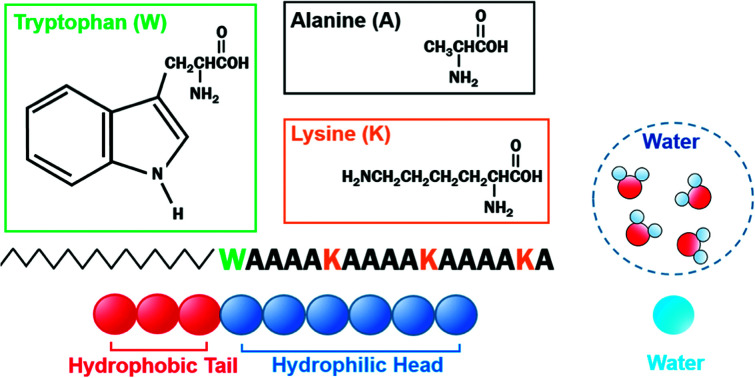

## 1. Introduction

Stimuli-responsive polymers are known to change their macroscopic properties based on variations in external conditions such as temperature, pH, light, and magnetic fields. Because of their unique nature, polymers have gathered great interest in academia and industry.^1^ Polymers exhibiting temperature-induced gelation characteristics have specifically attracted significant interest in the biological and biomedical fields.^2^ There are numerous reports on thermosensitive polymers having been created from poly(ethylene glycol),^3^ poly(ethylene oxide),^4^ and poly(*N*-isopropylacrylamide).^1,5,6^

Peptide amphiphiles (PA) have also attracted attention as another thermosensitive material.^7,8^ Amphiphilic molecules self-assemble to form a highly ordered nanoscale configuration in the presence of a solvent. Though general amphiphilic molecules lose their self-assembling ability and disperse into the solvent as temperature rises, some PAs have been found to exhibit temperature-induced gelation characteristics.^9,10^ The amphiphiles also show excellent biocompatibility and biodegradability, making them one of the most prospective materials for drug delivery systems,^11^ scaffolds for tissue engineering,^12^ regenerative medicine,^13^ and other “tailor-made” materials for biomedical use.^14^

Besides exploring new sequences to inject functionality into PA for particular applications, considerable effort has been put into understanding the self-assembly mechanism. Several studies have revealed that the self-assembly mechanism of PA is more complicated compared to conventional amphiphilic molecules because their assembly behavior is extensively affected by the secondary structure of α-helix and β-sheet transitions.^12,15–18^ Nowak *et al.*^12^ employed rheological and morphological analyses on PA with several peptide sequences and found that gelation was tied to the conformation of the hydrophobic peptide domains. They reported that the α-helical segments worked as good gelators, followed by the β-sheet and then the random coils. Such simultaneous phase transitions were also reported by Ding *et al.*,^15^ which had conducted both experimental observations and a coarse-grained molecular dynamics simulation using a simulator called COGNAC,^19^ to confirm that the self-assembly transition from an α-helix to a random coil would cause a change in the micelle structure from a worm to a sphere. Furthermore, Lee *et al.*^16^ conducted an atomistic molecular dynamics simulation on PA to construct nanofibers and found that water and ions could still penetrate the outer core region even after the nanostructure became matured. Lee *et al.*^17^ also used a coarse-grained force field called MARTINI^20^ to perform simulations on PAs as long as a few microseconds and visualized the three-dimensional network transforming into a quasi-one-dimensional nanofiber. Fu *et al.*^18^ used ePRIME,^21^ which is an extended PRIME model,^22^ as their coarse-grained model to simulate and adjusted the hydrophobicity to observe the various configurational transitions from open structures to a β-sheet and/or random coils.

We have worked with a particular PA named C_16_-WA_4_KA_4_KA_4_KA (hereafter C16-W3K), which consists of a hydrophobic alkyl chain and hydrophilic peptides containing tryptophan (W), lysine (K), and alanine (A).^23–25^ Their aqueous solution revealed that the self-assembly structure transition from a spherical to a worm-like micelle and peptide conformation transitions from α-helix to β-sheet simultaneously took place with macroscopic sol–gel transitions, which was found to be temperature-sensitive. The reproduction of the experiments took nearly a month at room temperature but was significantly accelerated at 50 °C, where the product could be made in 90 minutes. It was also noteworthy that the worm-like micelle structure could be maintained even in dry conditions, whereas self-assembled structures of other general amphiphilic molecules were produced only in the presence of a solvent. Such high stability of the worm-like micelle should be largely due to the contribution of the intermolecular hydrogen bonds of the β-sheets. The precise mechanism of the phase transitions of C16-W3K has yet to be elucidated, but Zhou *et al.*^4^ explained that the source of thermosensitivity of hyperbranched poly(3-ethyl-3-oxetanemethanol)–poly(ethylene oxide) (HBPO-*star*-PEO) was from the hydrogen bonding ability of a PEO segment weakening as temperature rose, leading to a partial collapse of the hydration shell around the aggregates. The disruption of the hydration shell induced the collision and fusion of the aggregates resulting in a morphological transformation. Besides the previous experimental studies, Duce *et al.*^26^ found that inter-peptide hydrogen bonds limited the diffusivity and that the conformation eventually determined the morphology of the PA aggregates analyzed by molecular simulations. The concern with the molecular simulations, especially with the coarse-grained simulations, should be in establishing a scientific basis for the interaction parameters to support the findings.

In this study, we used experimental measurements, where we applied the Flory–Huggins theory to extract the interaction parameter for our coarse-grained model and applied it to dissipative particle dynamics (DPD) simulations.^27,28^ DPD is known to directly allocate molecular information to physical parameters such as the Flory–Huggins *χ* parameter,^29^ and numerous examples on the self-assembly of amphiphiles were successfully reproduced by this simulation method.^30–35^ Jury *et al.*^32^ constructed a coarse-grained model for C12E6, which is a non-ionic amphiphile, and produced a temperature-concentration phase diagram that was consistent with experiments. Nakamura *et al.*^35^ used the aforementioned C12E6 model and studied how the change in the interaction between the hydrophilic part and solution would affect the temperature–concentration phase diagram, whereas Arai *et al.*^33^ investigated the self-assembly dynamic mechanism of the configurational transformation from spherical to worm-like micelles.

There is still, however, limited work regarding the temperature effects on the self-assembly of PA using DPD, so both experimental and computational methods were used to further investigate our previous report on temperature-sensitive self-assembly of PA.^23^ The ensuing section will introduce experimental and simulation theories applied to the analysis of this study. The conditions for both methods will be explained, which will be followed by the results and discussion of this work.

## 2. Theory

### 2.1. Vapor pressure osmometry

Vapor pressure osmometry (VPO) measurements were used to calculate *χ* between the peptide segments and water. VPO is a quick and convenient method to measure thermodynamic properties for dilute and semi-dilute solutions of polymer with low molecular weight.^36,37^ The apparatus is composed of two thermistors that form two arms of a Wheatstone bridge in an enclosed measuring chamber. The temperature is carefully controlled so that the chamber is saturated with solvent vapor. Under these conditions, the pressure difference between a pure solvent and solution is obtained from the voltage (temperature) differences between the two thermistors when a drop of pure solvent and solution comes into contact with each thermistor. The measured voltage difference has the following relationship with the osmotic pressure Π and temperature *T*:1
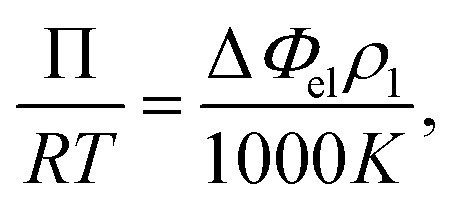
where Δ*Φ*_el_ is the voltage difference, *ρ*_1_ is the density of solvent, *R* is the gas constant, and *K* is a calibration constant. The chemical potential related to the solvent activity in the solution and the osmotic pressure is:

2 Δ*μ*_1_ = *RT* ln *a*_1_ = −*ν*_1_Π.

According to the Flory–Huggins theory,^38^ the interaction between a solvent and solute can be calculated from3
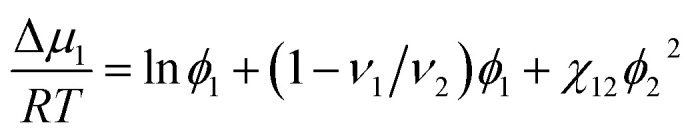
where *ν*_1_/*ν*_2_ ≈ 0 is the ratio of the molar volumes of the solvent and polymer. Moreover, the volume fractions of the solvent and polymer are denoted by *ϕ*_1_ and *ϕ*_2_, respectively. This equation could be rewritten as:4
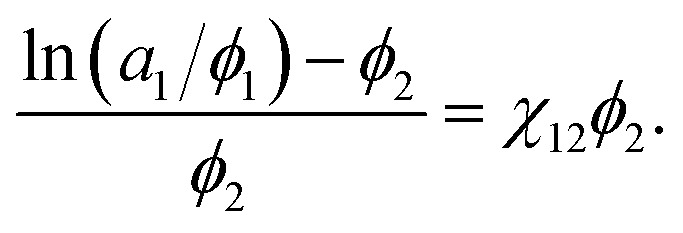


If the left-hand side of eqn (4) is plotted against *ϕ*_2_, the solvent/solute interaction parameter *χ*_12_ can be obtained from the slope of the fitted line.

### 2.2. Dissipative particle dynamics

The DPD method^27,29^ is based on Newton's equation of motion for a particle *i*,5
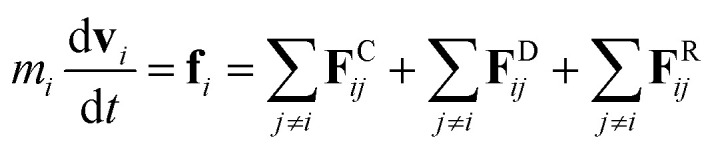
where *m* is the mass, **v** is the velocity, **f** is the total force and **F**^C^, **F**^D^, **F**^R^ are the conservative, dissipative, and random forces, respectively, each of which is given by:6
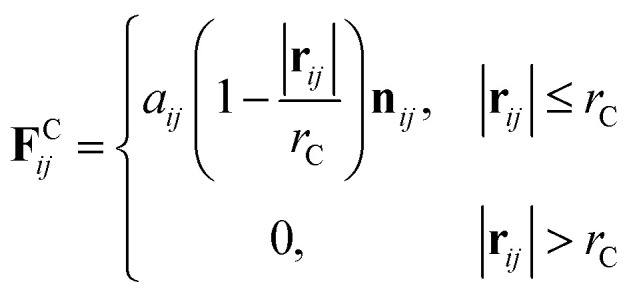
7
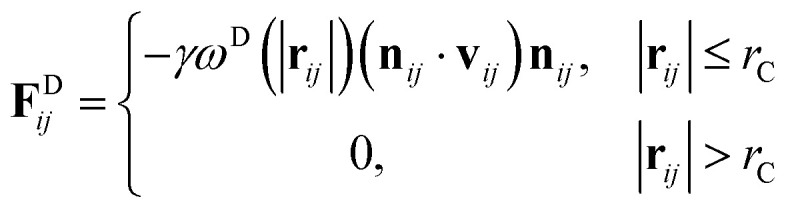
8
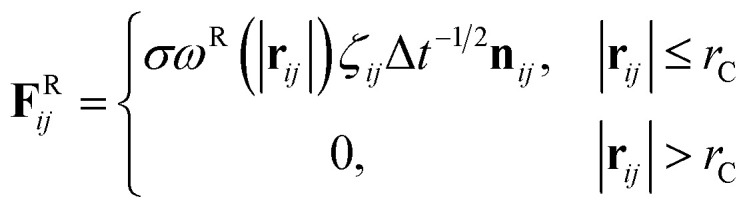
where *a*_*ij*_ is the repulsion parameter between particles *i* and *j*. Additionally, *r*_C_ is the cutoff distance, *σ* and *γ* are the noise and friction parameters, respectively, *ξ* is a random fluctuating variable, and finally, *ω*^R^ and *ω*^D^ are weight functions that depend on *r* = |**r**_*ij*_|. Moreover, **v**_*ij*_ = **v**_*i*_ − **v**_*j*_, **n**_*ij*_ = **r**_*ij*_/|**r**_*ij*_| and *ω*^R^, *ω*^D^, as well as *σ* and *γ* have the following relations^39^9
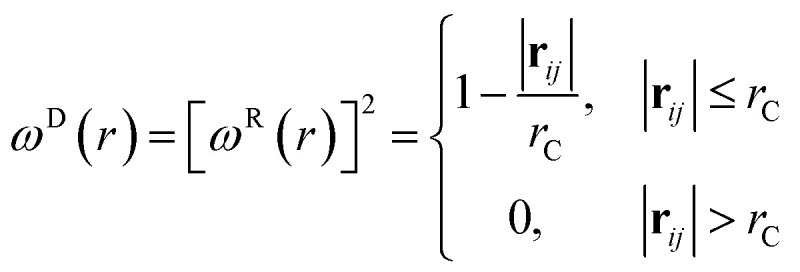

10 *σ*^2^ = 2*γk*_B_*T*

In addition, a spring force **F**^S^ shown below for the bond between DPD particles in PA is applied:11
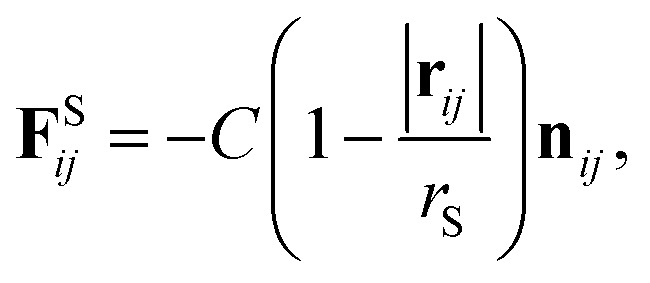
where *r*_S_ is the equilibrium bond distance and *C* is the spring constant.

## 3. Setup conditions

### 3.1. Experiment

#### 3.1.1. Preparation of the PA solution

Peptide W3K was synthesized by Scrum Inc. and treated with trifluoroacetic acid and piperidine to remove BOC- and FMOC-groups.^40,41^ The deprotected peptides were purified and freeze-dried before solubilizing in a distilled water at a concentration of ∼1–2 wt%. The characteristics of the deprotected peptides were verified by MALDI-TOF mass spectrometry.

#### 3.1.2. VPO measurement method

A vapor pressure osmometer manufactured by Knauer was used to measure the activity of water in the aqueous peptide solutions. The measuring chamber was filled with approximately 20 ml of pure solvent and then equilibrated at specific temperatures for a minimum of 8 hours for each experiment. Before the measurements, NaCl (Sigma-Aldrich Japan) was applied to calibrate the instrument. The Δ*Φ*_el_ values obtained for four different NaCl aqueous solutions (0.2–1 wt%) were separated by concentration before being plotted. The data points were linearly fit to acquire the calibration constant *K* in eqn (1), which was an extrapolated value of Δ*Φ*_el_/*c*, where *c* is the concentration. Finally, the estimated *K* values were 161 at 40 °C, 173 at 50 °C, and 194 at 60 °C.

The measurements for the aqueous peptide systems were performed for three different concentrated solutions ranging from 1 to 2 wt%. The temperature was varied from 40 °C to 60 °C because the manufacturer suggested the experiments to be carried out at a temperature that is at least 15 °C higher than room temperature for better stability and accuracy of the experiments.

### 3.2. Simulation method

**Fig. 1:**
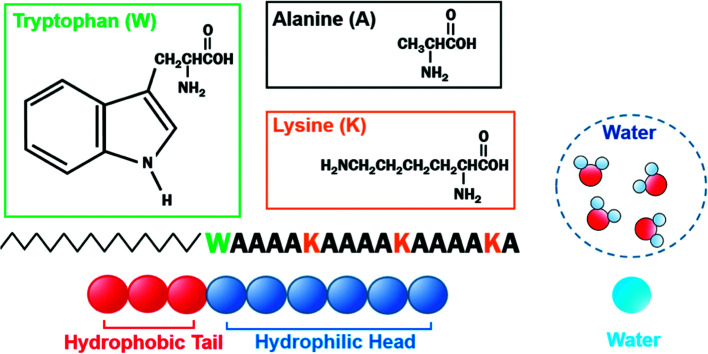
Schematic of coarse-grained C16-W3K and water. C16-W3K consists of an alkyl chain, which has a hydrophobic and a hydrophilic part. Three beads were used to represent the former, whereas six beads were incorporated for the latter. Four water molecules are described by a single bead.

The velocity Verlet algorithm was used to integrate the stochastic equations of motion into DPD^29^ through the OCTA platform.^19,42^ The PA model consists of nine bead-chain particles, where the first six in sequence had hydrophilic parameters representing the peptide head and the latter three represented hydrophobic tails that mimicked the alkyl chains as in Fig. 1. Groot and Warren^29^ connected the Flory–Huggins theory with DPD for a system with particle number 2 < *N* < 10 and *N*_A_ = *N*_B_, but there are numerous reports on variants that were also confirmed experimentally.^43–45^ In this system, the *N*_A_ : *N*_B_ ratio was determined from examining the actual length of peptide amphiphiles, where the head and tail had an actual length ratio of around 2 : 1; therefore, 6 : 3 was chosen because it falls within 2 < *N* < 10 and can reproduce the flexibility of the PA. The *a*_*ij*_ parameters in eqn (6) were decided from Yamamoto and Hyodo.^46^ The peptide-water interaction parameter *a*_HW_ was varied from 10 to 30 to describe the change in the hydration force, where the repulsive hydration force between hydrophilic peptides decreased as temperature increased.^4^ Other interaction parameters were predetermined to be *a*_HH_ = *a*_TT_ = *a*_WW_ = 25, *a*_HT_ = 40, and *a*_TW_ = 80, where W, H, and T denoted water, hydrophilic head, and hydrophobic tail, respectively. The total number of particles was constant at 18 000 and the volume fraction (*i.e.* the ratio of the number of particles) of the PA molecules was changed from 10% to 50%. For lower volume fractions of PA at 2% or 5%, larger systems of 135 000 and 72 000 particles were simulated, respectively, so there was always a sufficient amount of PA molecules within the system (Table 1). The basis of the number of particles was from test cases that found the worm-like micelle to be stable when it consists of around 200 chains (PA molecules). This is consistent with Arai *et al.*,^33^ where the number of molecules that form worm-like micelles were found to be around 160. Calculations were carried out for 500 000 steps at Δ*t* = 0.05*τ*, where *τ* is unit dimensionless time. During all simulations, the particle density *ρ* was 3.0 and the volume of the simulation box in dimensionless units was 18.1712^3^ for 10–50%, 28.8450^3^ for 5%, and 35.5689^3^ for 2%. The equilibrium bond distance *r*_S_ between particles in PA was set to 0.86 and the spring constant *C* was 4 based on earlier studies.^47–51^ Here, 0.86 corresponds to the average distance of the nearest neighbor particles. The effect of periodic boundary conditions has been examined in a separate study by Arai *et al.*^48^ Under the current system sizes that were examined, the percolation characteristics showed no change in the self-assembly configuration and minimal variation in the *a*_HW_ value. Equilibration was determined by comparing the results from 500 000 steps of four independent simulations from different initial coordinates. The results presented are only those that have produced the same terminal states.

**Table 1:** Concentration, number of particles, and box size in DPD simulation

Concentration [%]	Total number of particles	Number of PA molecules	Number of water particles	Box size [—]
2	135 000	300	132 300	35.5689^3^
5	72 000	400	68 400	28.8450^3^
10	18 000	200	16 200	18.1712^3^
15	18 000	300	15 300	18.1712^3^
20	18 000	400	14 400	18.1712^3^
30	18 000	600	12 600	18.1712^3^
40	18 000	800	10 800	18.1712^3^
50	18 000	1000	9000	18.1712^3^

## 4. Results and discussion

### 4.1. VPO measurements

**Fig. 2:**
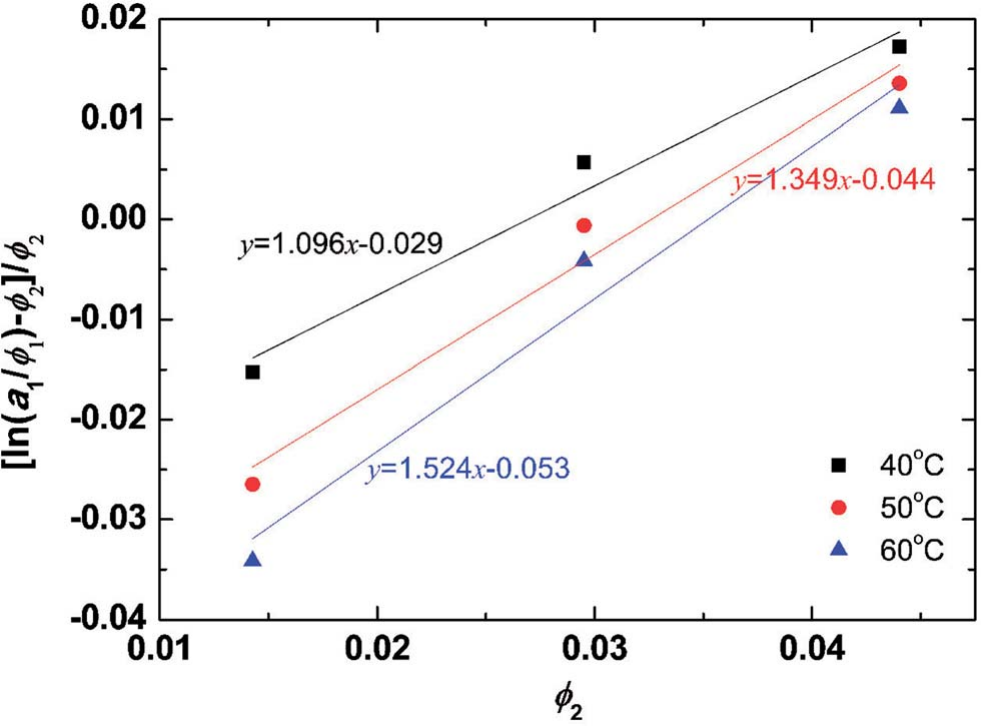
Activity dependence on the PA fraction of aqueous PA solution and temperature from the Flory–Huggins theory. To confirm the validity of the fitting, we have calculated the correlation coefficient for each condition. As a result, the correlation coefficient for 40, 50, 60 °C became 0.9884, 0.9884, and 0.9853, respectively. For sample size *n* = 3, significance level *α* = 0.1 the critical value is 0.9877, and for *n* = 3, *α* = 0.12 the critical value is 0.9823. Therefore, the data for 40 and 50 °C is true for a significance level of 10% and for 60 °C the correlation is true for a significance level of 12%.

The solvent activities were measured by VPO for the aqueous solutions of the peptides at concentrations of 1–2 wt%. The experimental results were plotted in Fig. 2 using eqn (4) from the Flory–Huggins theory. The volume fraction of peptides *ϕ* was 2900 ml mol^−1^ from density measurements. A linear fit was used in the VPO measurements, which was inserted into eqn (4). The corresponding *χ* values were 1.10 at 40 °C, 1.35 at 50 °C, and 1.52 at 60 °C, indicating that the interaction between the peptides and water became more repulsive as the temperature rose. Based on these results, the temperature dependence of *χ* for the peptides was found. Furthermore, the Flory–Huggins *χ* parameter is also known to have a relation with the interaction parameter *a*_*ij*_ as in eqn (26) and (27) of ref. 29.

12 *a*_*ij*_(*T*) = *a*_*ii*_ + Δ*a*(*T*)

13 Δ*a*(*T*) ≈ 3.268*χ*(*T*)

The *a*_HW_ value is calculated from eqn (12) by inserting 25 for *a*_*ii*_ and the aforementioned *χ* values providing a maximum of *a*_HW_ = 30, which is used in the simulations. A 10 °C difference produces an approximately 0.65 variation in *a*_HW_.

### 4.2. DPD simulations

**Fig. 3:**
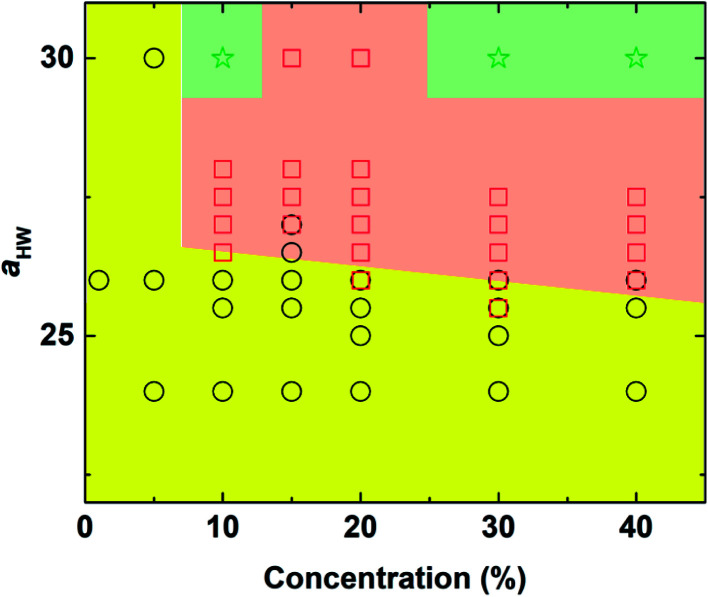
Concentration-*a*_HW_ phase diagram determined by DPD simulation. Circle: spherical micelles, square: worm-like micelles, and star: phase separation (worm-like micelles spanned across the system).

The phase diagram with concentration and the *a*_HW_ parameter is plotted in Fig. 3. As the concentration of PA and *a*_HW_ increased, a transition in the self-assembly structures changed from spherical to worm-like micelles. At higher concentrations with *a*_HW_ exceeding 30, the worm-like micelles spanned across the system, which resembled phase separation. These results are consistent with the works of Nakamura and Tamura,^35^ which reported a macroscopic phase transition of a dimer model starting from the hexagonal phase changing to the micellar phase with a complete phase separation occurring at the end. This phase transition phenomenon is similar to the lower critical solution temperature transitions reported for amphiphilic copolymers.^4^ Lee *et al.*^17^ also reported PA micelles changing into a branched-fiber structure across the periodic system. The phase separation found from the simulations, however, could not be observed in our PA experiments, since it was found that the C16-W3K molecules in our experiments severely degraded at temperatures around 70 °C. In terms of the *a*_HW_-dependence, it is clear from the figure that the critical concentration for the formation of worm-like micelles decreased in general as *a*_HW_ increased as seen in Fig. 3.

**Fig. 4:**
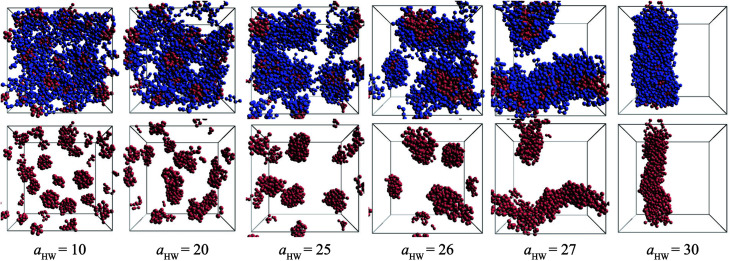
Snapshots at *t* = 25 000*τ* for various *a*_HW_ at concentration 15%. The upper row shows both hydrophilic (blue) and hydrophobic (red) groups, whereas the lower row only shows the latter.

Snapshots taken at *t* = 25 000*τ* for *a*_HW_ ranging from 10 to 30 at a concentration of 15% are presented in Fig. 4. The hydrophilic heads and hydrophobic tails of the PA model were described as blue and red beads, respectively in the panels above, and the lower figures represent the core of the micelles elongating with increasing *a*_HW_ (temperature). Water molecules were omitted for clarity in all figures. It is clear that the size of the spherical micelles became larger as *a*_HW_ increased. The shape of the micelles transformed into more worm-like structures rather than spheres when *a*_HW_ reached 26, and worm-like micelles were eventually observable when *a*_HW_ was above 27.

To characterize the self-assembly structure of PA more quantitatively, the asphericity of the micelles was calculated. When a coordinate system having an origin at the center-of-mass of a micelle with three principal axes of inertia is introduced, the asphericity *A* is defined as:^52,53^14
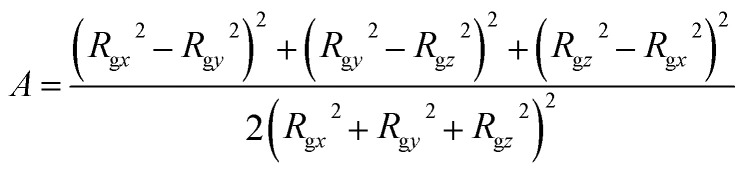
where *R*_g*x*_, *R*_g*y*_, and *R*_g*z*_ are the radii of gyration parallel to each principal axis.

**Fig. 5:**
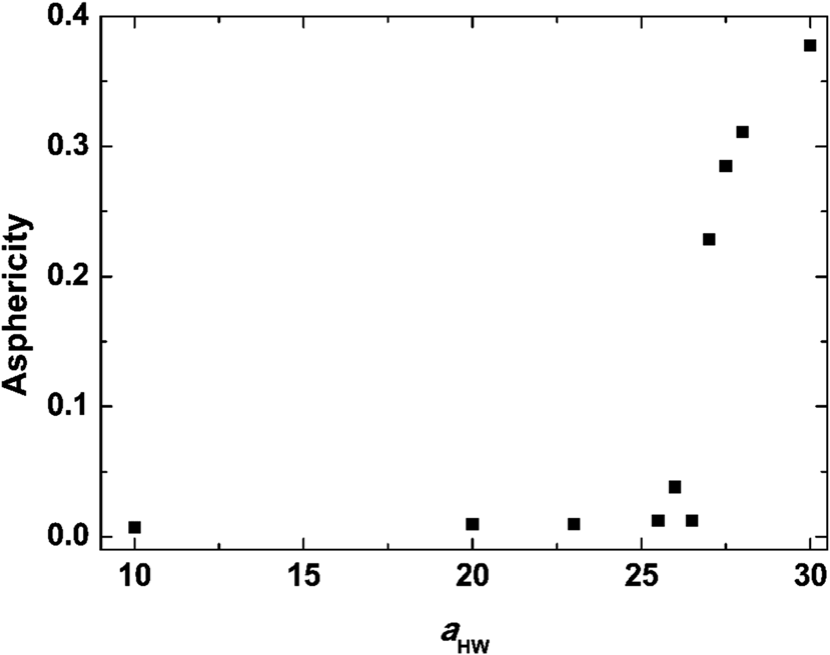
Asphericity of micelles observed at *t* = 25 000*τ* as a function of *a*_HW_ at concentration 15%.

Asphericity indicates the degree of deformation from spherical symmetry, where it is 0 for a perfect sphere and 1 for an infinite cylinder. The mean value of *A* for all micelles forming at *t* = 25 000*τ* as a function of *a*_HW_ is shown in Fig. 5 for a concentration of 15%. *A* was nearly 0 when *a*_HW_ was lower than 26.5, whereas a significant increase was observed for *a*_HW_ = 27, indicating a structural transition from spherical to worm-like had occurred.

**Fig. 6:**
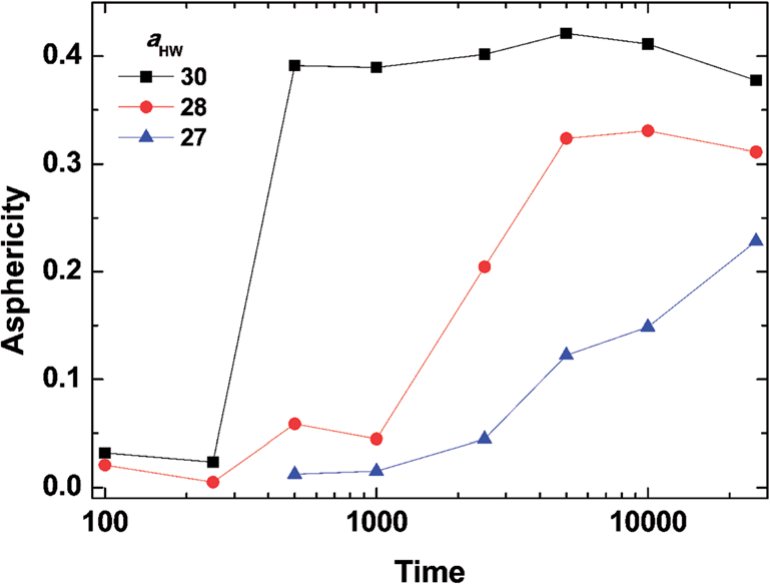
Asphericity evolution of micelles at concentration 15%. The lines are there to guide the eye.

To investigate the effect of *a*_HW_ on PA self-assembly over time, the mean values of *A* for the concentration of 15% with *a*_HW_ above 27 were taken from Fig. 4 and 5, and calculated as a function of time. Fig. 6 shows the results of the time evolution of *A*. It is clear that the transition time is highly dependent on *a*_HW_. When *a*_HW_ was 30, the transition ended 40 times faster compared to the transition at *a*_HW_ = 27. It is also noteworthy that the gradient of the asphericity became steeper. From these results, one can see that *a*_HW_ affects not only the eventual self-assembly structure of PA, but also the rate of self-assembly. Comparing these simulation results and our previous experimental reports,^23,25,54^ the *a*_HW_ dependence in simulations and temperature in experiments on the PA self-assembly coincided as expected from eqn (12). This is consistent with the findings from Shimada *et al.*,^55^ where experimentally, the C16-W3K solution will transition from a spherical micellar system to a worm-like system around 50 °C. This temperature corresponds to around *a*_HW_ = 26.5 for the DPD simulations at 15% concentration. The time evolution of the asphericity and temperature effect between the simulations and experiments are especially in accordance.

**Fig. 7:**
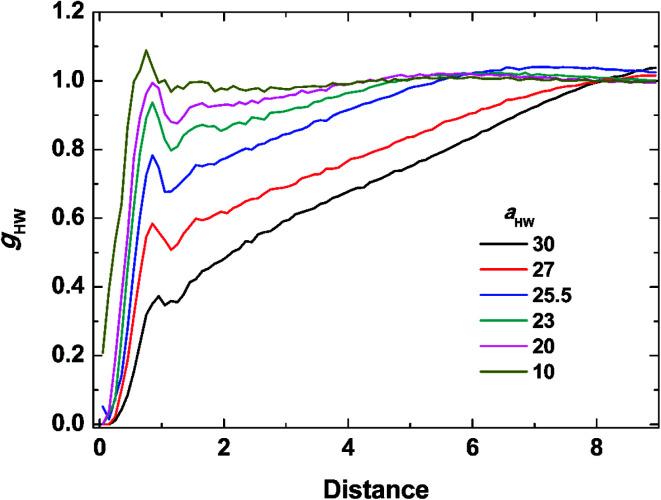
Pair distribution function for the peptides and water at concentration 15% and *t* = 25 000*τ*.

**Fig. 8:**
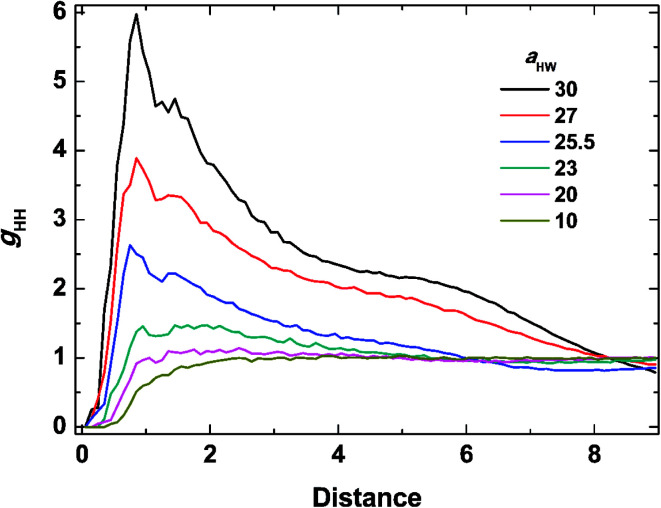
Pair distribution function for inter-molecular peptides at concentration 15% and *t* = 25 000*τ*.

To investigate the molecular structure in more detail, intermolecular pair-distribution functions *g*_HW_ and *g*_HH_ were plotted in Fig. 7 and 8, where *g*_HW_ represents the pair-distribution function for the peptides and water, and *g*_HH_ is for the intermolecular peptides. In Fig. 7, the value of *g*_HW_ stayed almost the same for *a*_HW_ = 10 but presented a significant decrease near the peptide as *a*_HW_ increased. This is because micelles grew with increasing *a*_HW_, thus pushing water away from the core, which was consistent with previous findings related to the hydration of peptides.^56,57^ Unlike Fig. 7, a sharp peak at a distance of 1 was observed as *a*_HW_ increased in Fig. 8. This peak indicates that the peptides merged at a constant distance from each other, and the amount of aggregation was proportional to the *a*_HW_ value. The variation in the packing of the peptides occurred irrespective of the interpeptide interaction parameter *a*_HH_. This is different from that found by Nakamura *et al.*,^35^ where *a*_HH_ was the parameter that directly affected the packing of the amphiphiles (especially the optimal surface area of the headgroup), whereas *a*_HW_ only indirectly influenced the packing. However, in our current results, *a*_HW_ showed a significant impact on the self-assembly behavior, showing a large contribution to peptide hydration. This difference is most likely due to the fact that Nakamura *et al.*^35^ used a non-ionic amphiphile as opposed to the zwitterionic peptide amphiphile used in this work. The fact that the difference in the innate hydrophilicities in the model changing the resultant interaction characteristics is natural.

From the simulation results, the effects of temperature, through *a*_HW_, on the mechanism of PA self-assembly can be understood. At high temperatures (with large *a*_HW_), the interaction between hydrophilic peptides and water became more repulsive. The relative repulsion from the peptides against water instead pulled other peptides towards themselves. Due to this attractive inter-peptide force, spherical micelles could easily gather closely, so they could fuse into worm-like micelles. Fast progress of phase transitions at high temperatures can be considered as a result of a higher probability of micelle fusion. On the other hand, at low temperatures, which could be represented by a low *a*_HW_ value, the interaction between hydrophilic peptides and water became relatively attractive. Therefore, water was distributed stably around the hydrated peptides, suppressing the aggregation of spherical micelles without further transitions into worm-like micelles. Such a slow progress of the phase transition at low temperatures could be regarded as a result of the low probability of micelle fusion.

## 5. Conclusion

VPO measurements were conducted prior to DPD calculations to model the interaction parameters to simulate the self-assembly of PA dissolved in an aqueous solution. In terms of the Flory–Huggins theory, the interaction between the peptides and water was confirmed to be more repulsive as the temperature increased. The dynamics of the self-assembly and resulting microstructures of PA revealed to be highly dependent on the value of the repulsion parameter *a*_HW_ by DPD simulation, and the relation between temperature and repulsion parameter *a*_HW_ was clarified. The mechanism of temperature dependence on the phase transition of PA starts from water molecules hydrating the hydrophilic head groups at low temperature, leading to a low probability of micelle fusion. On the other hand, the interaction between hydrophilic head groups became relatively attractive at high temperatures, leading to a high probability of micelle fusion and thereafter worm-like transition.

The primary link between simulation and experiment in this study does not lie in conventional parametric fitting, but rather in attaining an understanding in the thermosensitivity of interactions between peptide amphiphiles in an aqueous solution through both methods simultaneously. The relation between an increase in temperature (experimental) and increase in hydrophobicity has become clear (computational). Based on this finding, we confirmed that *a*_HW_ plays an important role in self-assembly characteristics. The methodology in quantitatively linking the coarse-grained parameters from experiment and theory provides a sensible foundation for bridging future simulation studies with experimental work on macromolecules.

## Conflicts of interest

There are no conflicts of interest to declare.

## Supplementary Material
